# Supplementary material for: Self-assembly Controls Self-cleavage of HHR from ASBVd (−): a Combined SANS and Modeling Study
Source: Sci Rep. 2016 Jul 26;6:30287. doi: 10.1038/srep30287 (PMC4960562; doi:10.1038/srep30287)
Supplement: Supplementary Information [file srep30287-s1.pdf]

Self-assembly Controls Self-cleavage of HHR from ASBVd(-): a Combined SANS and Modeling Study.

Fabrice Leclerc[1,*], Giuseppe Zaccai[2,3,4,5], Jacques Vergne[6], Martina Řìhovà[7], Anne Martel[2], and Marie-Christine Maurel[8,*]

[1]Institute for Integrative Biology of the Cell (I2BC), Dept. of Genome Biology, CEA, CNRS, Université Paris Sud, Gif-sur-Yvette, F-91198, France
[2]Institut Laue Langevin, Grenoble, F-38042, France
[3]Institut de Biologie Structurale (IBS), CNRS, Grenoble, F-38044, France
[4]IBS, CEA, Grenoble, F-38044, France
[5]IBS, Univ Grenoble Alpes, Grenoble, F-38044, France
[6]Institut de Systématique, Evolution, Biodiversité ISyEB - UMR 7205 - CNRS, MNHN, UPMC, EPHE, UPMC, Sorbonne Universités, 57 rue Cuvier, CP 50 Paris, F-75005, France
[7]Institute of Physics, Charles University, Faculty of Mathematics and Physics, Prague 2, CZ-121 16, Czech Republic
[*]fabrice.leclerc@i2bc.paris-saclay.fr,marie-christine.maurel@upmc.fr

ABSTRACT

In the Avocado Sunblotch Viroid (ASBVd: 249-nt) from the Avsunviroidae family, a symmetric rolling-circle replication operates through an autocatalytic mechanism mediated by hammerhead ribozymes (HHR) embedded in both polarity strands. The concatenated multimeric ASBVd (+) and ASBVd (-) RNAs thus generated are processed by cleavage to unit-length where ASBVd (-) self-cleaves with more efficiency. Absolute scale small angle neutron scattering (SANS) revealed a temperature-dependent dimer association in both ASBVd (-) and its derived 79-nt HHR (-). A joint thermodynamic analysis of SANS and catalytic data indicates the rate-determining step corresponds to the dimer/monomer transition. 2D and 3D models of monomeric and dimeric HHR (-) suggest that the inter-molecular contacts stabilizing the dimer (between HI and HII domains) compete with the intra-molecular ones stabilizing the active conformation of the full-length HHR required for an efficient self-cleavage. Similar competing intra- and inter-molecular contacts are proposed in ASBVd (-) though with a remoter region from an extension of the HI domain.

Supplementary Information

ZIP archive (IntaRNA_Supp.zip) containing the calculations performed on the web server version of IntaRNA (http://bit.ly/IntaRNA) at the temperatures: 10 °C, 25 °C and 45 °C (see Materials and Methods for the other parameters).

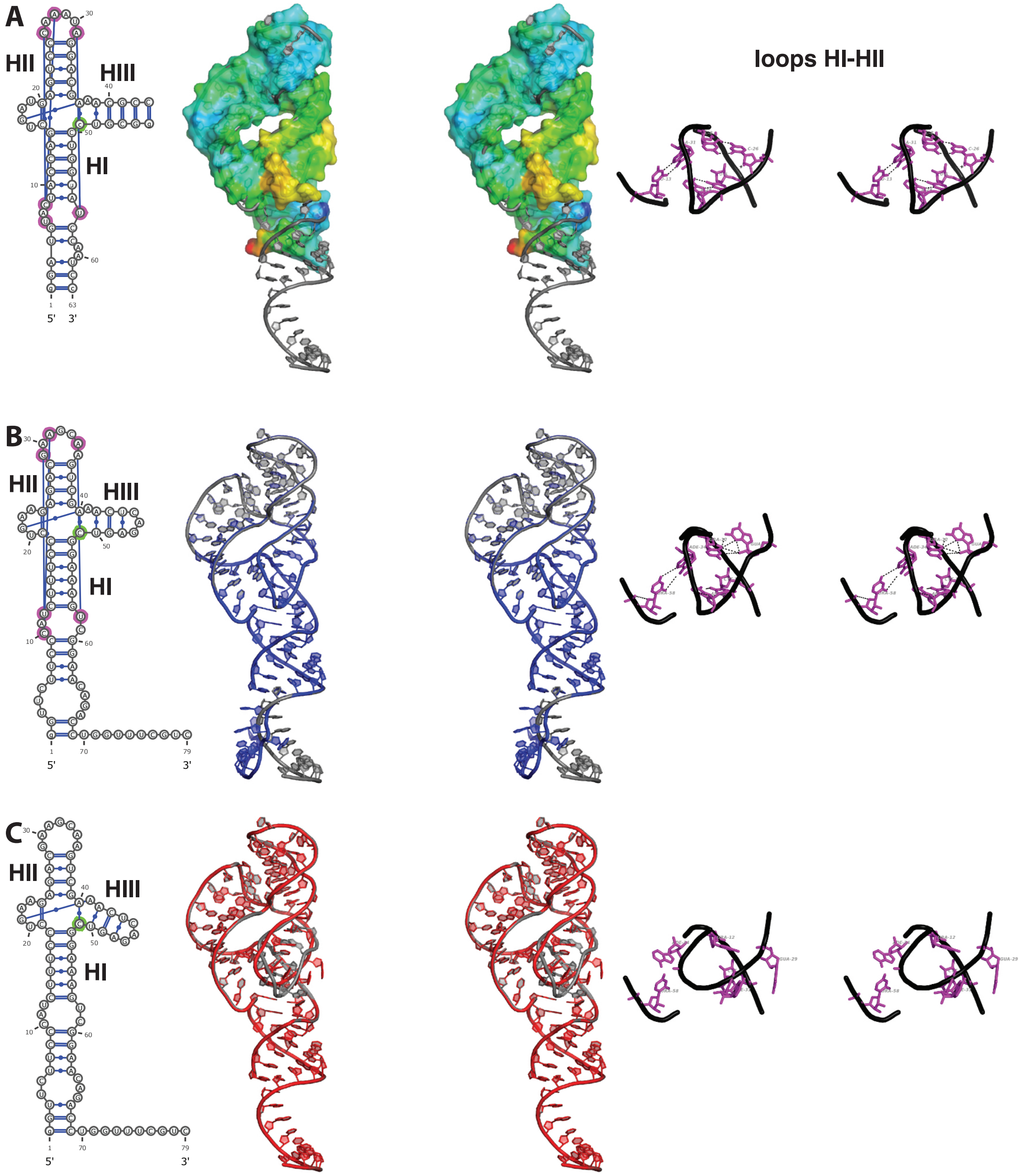

Supplementary Figure 1: Comparisons of RNA 3D Structures from X-ray crystallography and RNA Modeling. (Legend on the following page).

Supplementary Figure 1: Comparisons of RNA 3D Structures from X-ray crystallography and RNA Modeling.(A) 2D and 3D structures of the template and modeled ribozymes.Left: 2D structure of the template corresponding to the X-ray 3D structure of a minimal HHR ribozyme (PDB ID: 2OEU). Middle: stereo-view of the superposition between the 3D model of HHR (-) (79-mer) and the X-ray 3D structure of the template (PDB ID: 2OEU). The X-ray structure is represented by a solvent accessible surface using a color code corresponding to the B factors. The 3D model (grey) in cartoon representation shows slight structural differences: the presence of a triloop closing the HIII domain, the presence of bulge nucleotides in the HI domain which is also significantly extended in ASBVd. Right: stereo-view of the intramolecular tertiary contacts between the domains HI and HII in the template (PDB ID: 2OEU).(B) 2D and 3D structures of the foldedmonomer preserving tertiary contacts. Left: 2D structure with indication of the homologous 3D tertiary contacts. Middle: stereo-view of the superposition between the 3D model of HHR (-) in its unbound (grey)/bound (blue) conformations for the first monomer. Right: stereo-view of the intramolecular tertiary contacts between the domains HI and HII in the folded monomer.(C) 2D and 3D structures of the unfoldedmonomer in the HII terminal loop. Left:2D structure of the unfolded monomer.Middle: stereo-view of the superposition between the 3D model of HHR (-) in its unbound (grey)/bound (red) conformations for the unfolded monomer.Right: stereo-view of the lost intramolecular tertiary contacts between the domains HI and HII in the unfolded monomer.

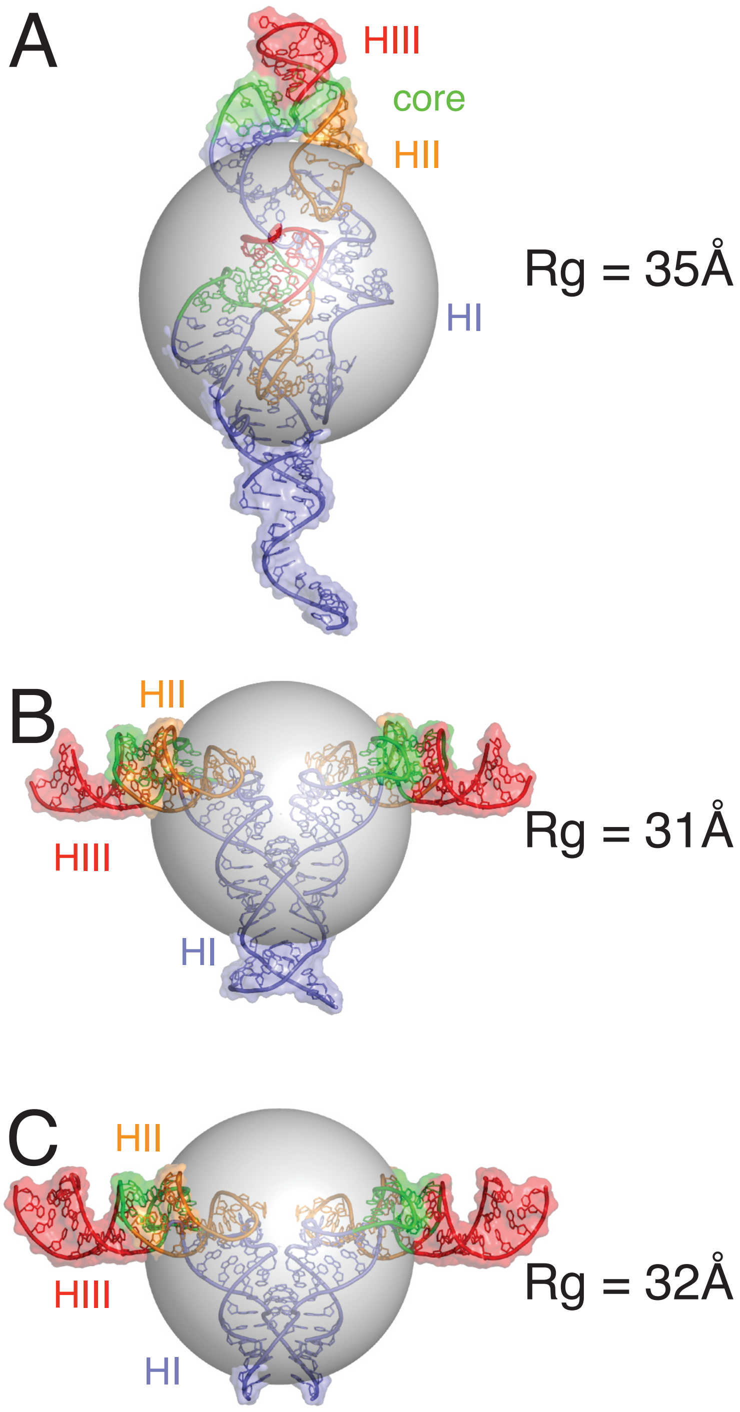

Supplementary Figure 2: Relationship between the mode of association and the radius of gyration of HHR dimers. (A) HI-HII mode of interaction in the 3D model of the HHR (-) dimer from ASBVd (Fig. 5C). (B) HI-HI mode of interaction in the 3D structure of an artificial HHR dimer with an extended HI domain (PDB ID: 5DI2, 68 nt + 11 nt). (C) HI-HI mode of interaction in the 3D structure of an artificial HHR dimer with an extended HIII domain (PDB ID: 5DI2, 68 nt + 11 nt). The 3D structures were extended by 11 nt in the HI or HIII domains to match the 79 nt length of HHR (-) and optimized using the same minimization protocol (see Methods). The HI, HII and HIII are colored in blue, orange and red, respectively; the three-way junction core is in green.

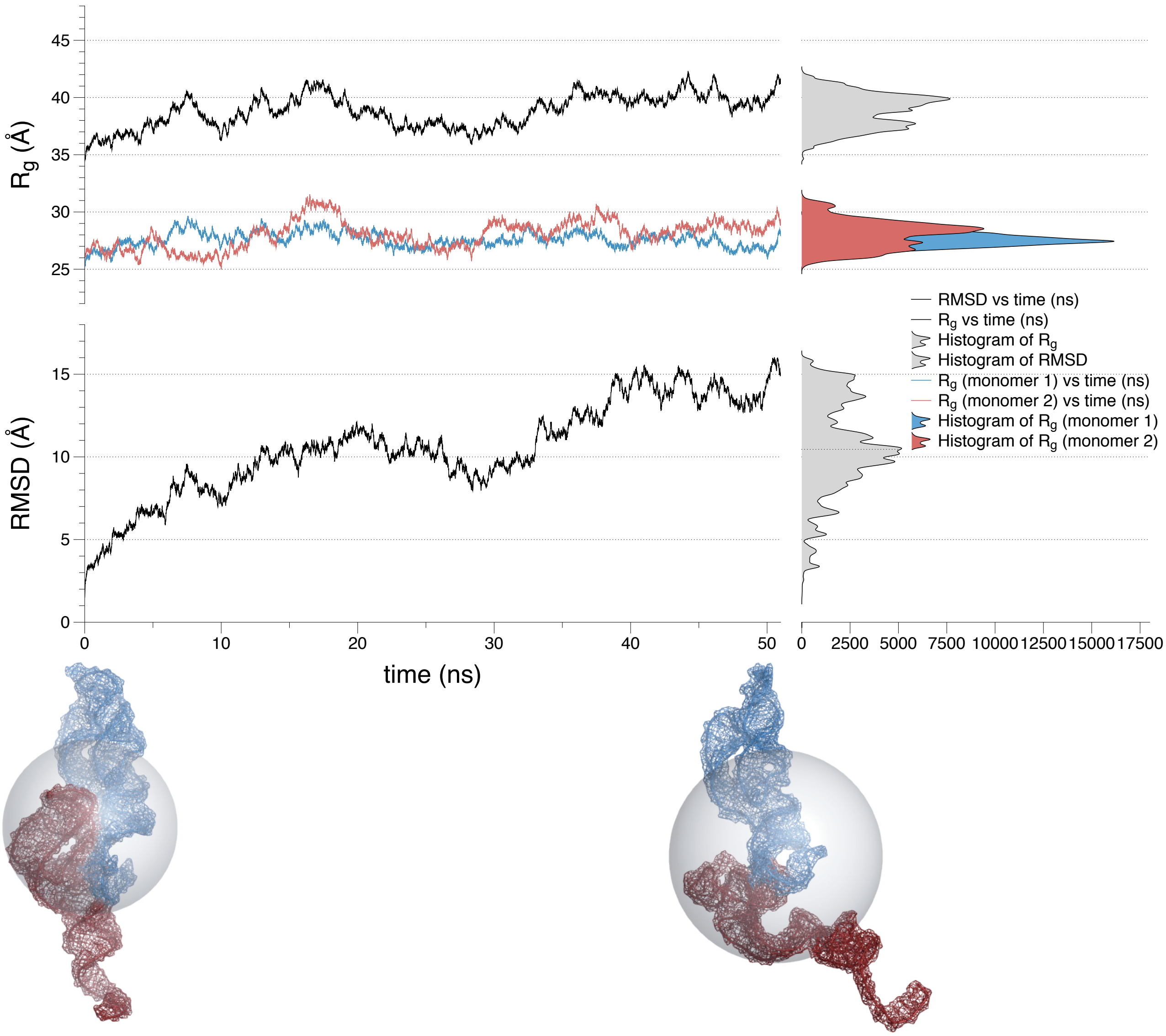

Supplementary Figure 3: Radius of gyration (R_g) and Root Mean Square Deviation ($RMSD$) versus time (ns). (Top) Time-dependent R_g and $RMSD$. The time-dependent of the dimer (black) and monomer R_g (monomer 1: blue; monomer 2: red) are shown. (Bottom) 3D conformations at the starting and ending points of the simulation (50ns): R_g^{start} = 35 Å (left) and R_g^{end} = 42 Å (right).
